# The glucocorticoid receptor gene (*NR3C1*) is linked to and associated with polycystic ovarian syndrome in Italian families

**DOI:** 10.1186/s13048-023-01329-5

**Published:** 2024-01-12

**Authors:** Shumail Syed, Claudia Gragnoli

**Affiliations:** 1 Division of Endocrinology, Department of Medicine, Creighton University School of Medicine, Omaha, NE 68124 USA; 2 Concord Hospital-Laconia, Laconia, NH 03246 USA; 3 Department of Public Health Sciences, Penn State College of Medicine, Hershey, PA 17033 USA; 4 Molecular Biology Laboratory, Bios Biotech Multi-Diagnostic Health Center, Rome, 00197 Italy

**Keywords:** Nuclear receptor subfamily 3 group C member 1, *NR3C1*, Glucocorticoid receptor, GR, Polycystic ovary syndrome, PCOS

## Abstract

**Objectives:**

Components of the hypothalamic-pituitary axis (HPA) pathway are potential mediators of the genetic risk of polycystic ovarian syndrome (PCOS). Impaired glucocorticoid receptor (*NR3C1*) expression and function may underlie impaired HPA-axis cortisol activity, thereby also contributing to the increased adrenal cortisol and androgen production present in women with PCOS. In this study, we aimed to identify whether *NR3C1* is linked or in linkage disequilibrium (LD), that is, linkage joint to association, with PCOS in Italian peninsular families.

**Method:**

In 212 Italian families with type 2 diabetes (T2D) from the Italian peninsula, previously recruited for a T2D study and phenotyped for PCOS, we used microarray to genotype 25 variants in the *NR3C1* gene. We analyzed the 25 *NR3C1* variants by Pseudomarker parametric linkage and LD analysis.

**Results:**

We found the novel implication in PCOS risk of two intronic variants located within the *NR3C1* gene (rs10482672 and rs11749561), thereby extending the phenotypic implication related to impaired glucocorticoid receptor.

**Conclusions:**

To the best of our knowledge, this is the first study to report *NR3C1* as a risk gene in PCOS.

**Supplementary Information:**

The online version contains supplementary material available at 10.1186/s13048-023-01329-5.

## Highlights

NR3C1 is a novel risk gene in polycystic ovarian syndrome.

NR3C1 appears to be implicated in polycystic ovarian syndrome through modulating the stress response.

**Supplementary Information:**

The online version contains supplementary material available at 10.1186/s13048-023-01329-5.

## Background

Polycystic ovarian syndrome (PCOS) is among the most common disorders in women of reproductive age, affecting up to 15% of women worldwide, depending on the diagnostic criteria used; it manifests as a heterogenous syndrome encompassing various combinations of otherwise unexplained hyperandrogenism, anovulation, and polycystic ovaries [1]. PCOS typically follows a polygenic inheritance pattern that is analogous to type 2 diabetes (T2D) and obesity, reflecting an interaction of susceptibility genes and environmental factors [1]. Clinical features include biochemical hyperandrogenism (e.g., elevated blood levels of testosterone and androstenedione), cutaneous signs of hyperandrogenism (e.g., hirsutism, moderate-to-severe acne, male-pattern baldness), menstrual irregularity, polycystic ovaries, insulin resistance, and obesity. The diagnosis is associated with increased risk of infertility, metabolic syndrome, T2D, and cardiovascular disease, as well as endometrial carcinoma [2, 3] and anxiety and depression [4]. In women with PCOS, the hypothalamic-pituitary adrenal (HPA) axis may be impaired [5], namely the corticotropin (CRH), adrenocorticotropin (ACTH), and cortisol pathway. In particular, research indicates an association between PCOS and enhanced adrenal sensitivity to ACTH [6].

A significant player of the cortisol pathway is the glucocorticoid receptor (NR3C1 or GR), which is encoded by the ubiquitously expressed *NR3C1* gene and mediates the HPA-axis negative feedback at the hypothalamus and pituitary level for the release of CRH and ACTH, respectively [7, 8]. Impaired glucocorticoid receptor expression (*NR3C1*) and function (NR3C1) may underlie the PCOS-related impaired HPA-axis cortisol feedback inhibition, thus contributing to the increased cortisol and adrenal androgen production occurring in women with PCOS [9]. More than 50% of women with PCOS have impaired glucocorticoid sensitivity [10], possibly due to NR3C1 resistance. Of note, PCOS is associated with increased serum NR3C1 protein concentration [11], which might explain both higher cortisolemia and decreased glucocorticoid sensitivity of PCOS by NR3C1 resistance. Variants in the *NR3C1* gene have been reported to contribute to metabolic syndrome in patients with PCOS [12] and to insulin resistance [13], which is a feature of PCOS indirectly contributing to increased free androgen blood levels via reduction of the sex-hormone binding globulin blood levels [14].

We recently reported *NR3C1* variants conferring pleiotropic risk effects in T2D and major depressive disorder (MDD) [15]. In the present study, we hypothesized that *NR3C1* might also contribute to PCOS, which manifests with both metabolic and mental traits. We aimed to investigate whether *NR3C1* is linked or in linkage disequilibrium (LD, i.e., linkage joint to association) with PCOS in Italian peninsular families.

## Materials and methods

We genotyped 25 variants in the *NR3C1* gene using microarray in 212 Italian families originated from the Italian peninsula and previously recruited for a T2D study (Supplementary Table 1). The patients were then later phenotyped for the presence or absence of PCOS following the Rotterdam diagnostic criteria (presence of at least two of the following three characteristics: chronic anovulation or oligomenorrhea, clinical or biological hyperandrogenism, and/or polycystic ovaries) [16]. To consider a subject positive for PCOS, thyroid hormonal impairments, hyperprolactinemia, hypothalamic amenorrhea, and congenital adrenal hyperplasia were excluded, and two or more of the following inclusion criteria needed to be present: chronic anovulation or oligomenorrhea, clinical or biochemical hyperandrogenism, and/or polycystic ovaries [16]. The amplified variants were first tested and excluded to have any Mendelian and genotyping errors using PLINK [17]. Further, the variants were then tested via Pseudomarker [18] parametric analysis for linkage and/or LD to/with PCOS according to the following models: dominant models with complete (D1) and incomplete penetrance (D2) and recessive models with complete penetrance (R1) and incomplete penetrance (R2). P values of < 0.05 were used as the cut off for statistical significance. The investigated variants were also tested for the presence or absence of LD blocks according to the correlation coefficient between SNPs by analyzing the Tuscany Italian population derived from the 1000 Genomes Project (https://www.internationalgenome.org/data-portal/population/TSI). The study was approved by the Bios Ethical Committee and written informed consent was obtained from each participant for enrollment in the study.

### In-silico analysis

We conducted *in-silico* analysis for detected risk variants predicted roles in transcription-factor binding (SNP2TFBS [19]), splicing (SNP-function prediction [20]), miRNA binding (mirSNP [21]), and interaction with chromatin state (RegulomeDB [22]).

## Results

### Patients

In the familial dataset with T2D, 11% of families were positive for PCOS. The PCOS patients’ average BMI at age 20 was 24.73 (range 19.53–34.08) with 30% being overweight (BMI ≥ 25) and 13% being obese (BMI ≥ 30). The PCOS patients’ average maximum lifetime BMI was 32.51 (range 20.57–69.85) with 74% being overweight (BMI ≥ 25) and 39% being obese (BMI ≥ 30). The average increment of BMI from BMI age 20 to maximum lifetime BMI was 1.36. The average difference between the maximum lifetime BMI and the BMI at age 20 was 8.91.

### Genetic findings

**Fig. 1:**
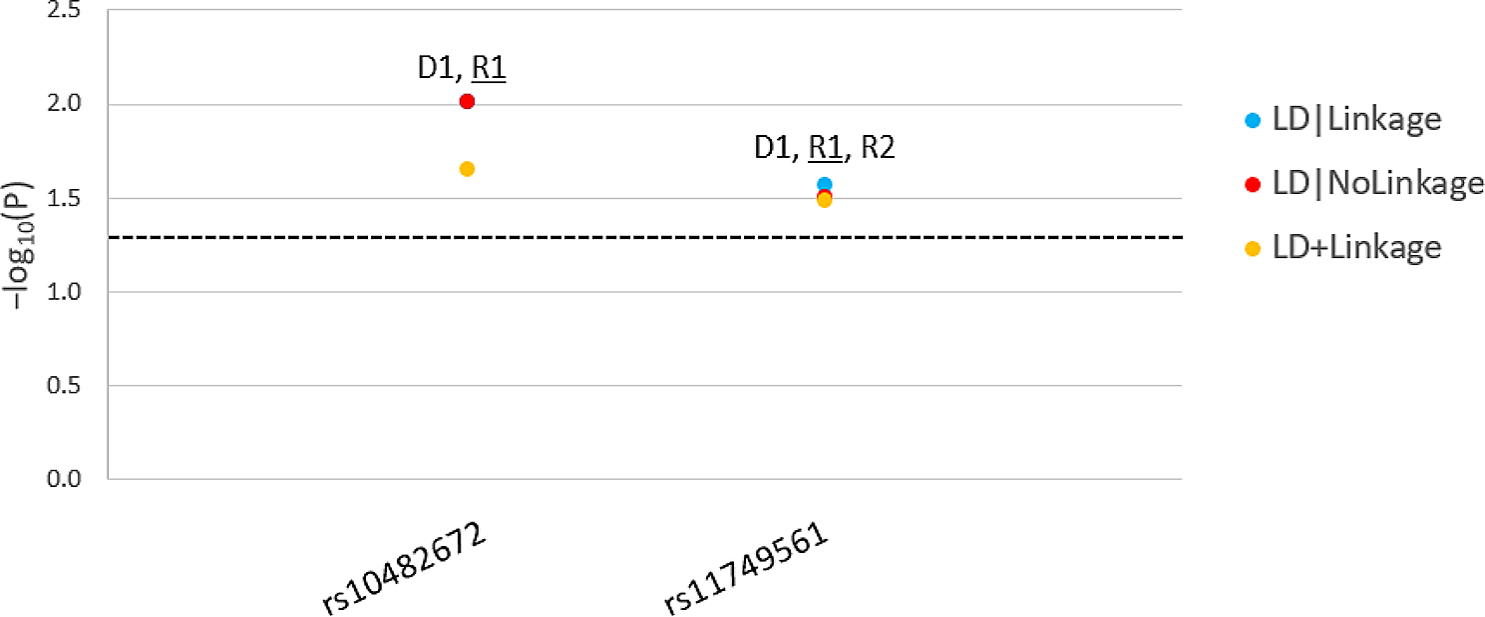
Parametric Analysis Results of Polycystic Ovarian Syndrome (PCOS) *NR3C1*-Risk Single Nucleotide Polymorphisms (SNPs). For each *NR3C1*-risk SNPs in PCOS, we present the − log10(P) as a function of the significant (P < 0.05) test statistics (linkage disequilibrium [LD]|Linkage, LD|No Linkage and LD + linkage) and per inheritance model. D1: dominant, complete penetrance, R1: recessive, complete penetrance, R2: recessive, incomplete penetrance. The most significant model is underlined

We identified two intronic variants (rs10482672 and rs11749561) that are significantly associated with the risk of PCOS (P < 0.05). These variants were significant across several inheritance models but more prominently under R1 (Fig. 1) suggesting that the risk is genotype-related rather than allelic. The two variants are novel (Table 1) and have not been previously associated with the risk of PCOS or any of its related phenotypes (i.e., metabolic syndrome, hyperglycemia, irregular menses, anovulation, infertility, oligomenorrhea, obesity, insulin resistance, T2D, hyperandrogenism, hirsutism). The two novel risk variants are intronic and *in-silico* functional predictions yielded no findings except for the intersection with repressed chromatin state in the ovarian tissue (RegulomeDB [22]).

**Table 1:** Polycystic Ovarian Syndrome (PCOS) *NR3C1*-Risk Single Nucleotide Polymorphisms (SNPs)

Model^1^	SNP	Position	Ref	Alt	Risk Allele	Consequence	LD block	Reported in PCOS or related phenotypes/traits?*
D1, R1	rs10482672	143312968	G	A	G	Intronic	Independent	Novel
D1, R1, R2	rs11749561	143412112	T	C	T	Intronic	NA	Novel

^1^Models: D1: dominant, complete penetrance, R1: recessive, complete penetrance, R2: recessive, incomplete penetrance. *The PCOS related phenotypes are: metabolic syndrome, hyperglycemia, irregular menses, anovulation, infertility, oligomenorrhea, obesity, insulin resistance, T2D, hyperandrogenism, hirsutism

## Discussion

The glucocorticoid receptor (NR3C1) is an essential component of the adaptive stress response [23]. Considering the versatility of pathologies related to stress maladaptation [24], components of the stress response have been implicated in several complex mental and metabolic disorders [25, 26]. The *NR3C1* gene in particular was previously implicated in the risk of comorbidity of T2D and MDD in the peninsular Italian families of the current study [15]. We now report the novel implication of the *NR3C1* gene in the risk of PCOS, which is also a complex disorder carrying increased risks for T2D and depression [27, 28]. In our previous study of familial T2D [15], the identified NR3C1 PCOS-risk SNPs were not found to confer risk for T2D, which would have underlined a T2D-PCOS comorbid risk for the above-mentioned detected PCOS variants.

Previous studies of *NR3C1* and PCOS or its endophenotypes were inconclusive, some case-control studies in similar ethnic populations (Caucasians) failed to detect significant association [29, 30], probably due to the lower detection power of sporadic cases compared to familial cases, while others were positive [12], though in a case-control cohort from a different ethnic population (Brazilian) and with the endophenotype of insulin resistance rather than PCOS itself.

We identified two novel intronic variants significantly linked to and associated with the risk of PCOS in peninsular Italian families. The rs11749561 variant was previously studied for association with weight gain induced by diabetes therapy, but no association was found [31]. The pathogenic mechanisms of the two risk variants reported in our study are yet to be determined. Our *in-silico* analysis yielded negative results, except for the two variants intersecting with repressed chromatin state in the ovarian tissue (RegulomeDB [22]) which indicates a potential negative role in gene expression and quantitative decrease in NR3C1 protein. Despite this finding being related only to the ovaries, it does not align with the reported systemic increased serum NR3C1 concentration in PCOS [11], thereby making functional studies necessary to validate these results. Furthermore, replication of our linkage and association finding in other ethnic groups is needed.

Our study has several limitations. First, the studied population is a homogenous monoethnic group which hinders the generalization of our findings. Second, our method of exploring the variations in the *NR3C1* gene is limited to the amplified variants, and direct sequencing of the *NR3C1* gene is warranted to explore nearby risk variants in LD to the identified risk variants in our study. And third, the functional role of NR3C1 in PCOS needs to be investigated. Therefore, in vitro and/or in vivo studies are needed in order to reach more solid conclusions.

## Conclusions

To the best of our knowledge, this is the first study to report *NR3C1* as a risk gene in PCOS.

### Electronic supplementary material

Below is the link to the electronic supplementary material.

**Supplementary Material 1: Supplementary Table 1.** Single nucleotide polymorphisms (SNPs) analyzed in PCOS

## Data Availability

The study data are available on reasonable request, and due to lacking specific patients’ consent and privacy restrictions, they are not publicly available.

## Declarations

### Authors’ information

C.G. is Professor of Medicine, Chief of Endocrinology, Endowed Puller Chair, Creighton University School of Medicine, Omaha, NE, and Adjunct Professor of Public Health Sciences, Penn State University College of Medicine, Hershey, PA; S.S. in an MD, graduated Endocrinology Fellow, Creighton University School of Medicine, Omaha, NE.
