# Supplementary material for: The glucocorticoid receptor gene (NR3C1) is linked to and associated with polycystic ovarian syndrome in Italian families
Source: J Ovarian Res. 2024 Jan 12;17:13. doi: 10.1186/s13048-023-01329-5 (PMC10785542; doi:10.1186/s13048-023-01329-5)
Supplement: Supplementary file 1 — Supplementary Material 1: Supplementary Table 1. Single nucleotide polymorphisms (SNPs) analyzed in PCOS [file 13048_2023_1329_MOESM1_ESM.pdf]

Supplementary Table 1. Single nucleotide polymorphisms (SNPs) analyzed in PCOS.					
**Chr**	**Position**	**SNP**	**REF**	**ALT**	**REF Frequency**
5	143280664	rs6197	A	G	0.017
5	143281925	rs6196	A	G	0.081
5	143282715	rs258751	G	A	0.006
5	143299858	rs10482681	A	C	0.031
5	143303272	rs115357812	A	G	0.028
5	143307929	rs852977	A	G	0.410
5	143308758	rs860457	T	C	0.421
5	143312968	**rs10482672**	G	A	**0.199**
5	143313762	rs10482668	T	A	0.005
5	143313891	rs10482667	T	C	0.005
5	143341028	rs72801072	A	G	0.005
5	143370968	rs10482633	T	G	0.290
5	143388175	rs9324921	C	A	0.103
5	143393242	rs75839648	G	A	0.006
5	143395855	rs62375507	C	T	0.018
5	143400155	rs72542742	C	T	0.002
5	143400647	-	A	C	0.004
5	143400772	rs6190	C	T	0.019
5	143400774	rs6189	C	T	0.022
5	143412112	**rs11749561**	T	C	**0.944**
5	143412919	rs9324924	G	T	0.460
5	143413085	rs7701443	A	G	0.767
5	143417706	rs12189168	A	G	0.016
5	143418420	rs13186836	T	C	0.414
5	143422369	rs13184611	C	T	0.412
**Legend.** Chr = Chromosome; REF = reference allele; ALT = alternative allele. The PCOS-risk SNPs and					

**ALT Frequency**	**Risk Allele**
0.983	G
0.919	A
0.994	G
0.969	C
0.972	G
0.590	G
0.579	C
0.801	G
0.995	T
0.995	T
0.995	G
0.710	G
0.897	A
0.994	A
0.982	C
0.998	C
0.996	C
0.981	C
0.978	T
0.056	T
0.540	G
0.233	G
0.984	A
0.586	C
0.588	T
